# Supplementary material for: Genetic evidence for the association between COVID-19 epidemic severity and timing of non-pharmaceutical interventions
Source: Nat Commun. 2021 Apr 12;12:2188. doi: 10.1038/s41467-021-22366-y (PMC8041850; doi:10.1038/s41467-021-22366-y)
Supplement: Supplementary file 1 — Supplementary Information [file 41467_2021_22366_MOESM1_ESM.pdf]

GENETIC EVIDENCE FOR THE ASSOCIATION BETWEEN COVID-19 EPIDEMIC SEVERITY AND TIMING OF NON-PHARMACEUTICAL INTERVENTIONS

Manon Ragonnet-Cronin*[1], Olivia Boyd[#1], Lily Geidelberg[#1], David Jorgensen[#1], Fabricia F. Nascimento[#1], Igor Siveroni[#1], Robert A. Johnson[1], Marc Baguelin[1], Zulma M Cucunubá[1], Elita Jauneikaite[1], Swapnil Mishra[1], Oliver J. Watson[1], Neil Ferguson[1], Anne Cori[1], Christl A. Donnelly[1,2], Erik Volz[1] on behalf of the Imperial COVID-19 Response Team

[1] MRC Centre for Global Infectious Disease Analysis and the Department of Infectious Disease Epidemiology, Imperial College London

[2] Department of Statistics, University of Oxford

SUPPLEMENTARY INFORMATION

Supplementary Tables

Supplementary Table 1 | Results for all linear regression models

Model	Formula	Predictor	Estimate	CI2.5	CI97.5	p	R2
1	log(nCases_maxNPI) ~ CESTtoNPI	CESTtoNPI	0.076	0.042	0.11	<0.0001	0.268
2	log(nCases_maxNPI) ~ log(popEstimateNearestThousand)	log(popEstimateNearestThousand)	0.638	0.262	1.013	0.0013	0.174
3	log(nCases_maxNPI) ~ case10toNPI	case10toNPI	0.087	0.046	0.129	<0.0001	0.243
4	log(nCases_maxNPI) ~ CESTtoNPI + log(popEstimateNearestThousand)	CESTtoNPI	0.061	0.024	0.098	0.0016	0.315
4	log(nCases_maxNPI) ~ CESTtoNPI + log(popEstimateNearestThousand)	log(popEstimateNearestThousand)	0.367	-0.015	0.749	0.0594	0.315
5	log(Ne) ~ CESTtoNPI	CESTtoNPI	0.052	0.031	0.074	<0.0001	0.316
6	log(Ne) ~ log(popEstimateNearestThousand)	log(popEstimateNearestThousand)	0.399	0.128	0.669	0.0046	0.147
7	log(Ne) ~ case10toNPI	case10toNPI	0.026	-0.004	0.056	0.0845	0.057
8	log(Ne) ~ log(popEstimateNearestThousand) + CESTtoNPI + R	log(popEstimateNearestThousand)	0.233	-0.042	0.508	0.0956	0.377
8	log(Ne) ~ log(popEstimateNearestThousand) + CESTtoNPI + R	CESTtoNPI	0.044	0.02	0.067	0.0005	0.377
8	log(Ne) ~ log(popEstimateNearestThousand) + CESTtoNPI + R	R	0.006	-0.001	0.013	0.0814	0.377
9	log(deaths) ~ CESTtoNPI	CESTtoNPI	0.049	0.012	0.086	0.0109	0.114
10	log(deaths) ~ log(popEstimateNearestThousand)	log(popEstimateNearestThousand)	0.35	-0.044	0.745	0.0807	0.055
11	log(deaths) ~ case10toNPI	case10toNPI	0.004	-0.043	0.051	0.8590	0.001
12	log(deaths) ~ CESTtoNPI + log(popEstimateNearestThousand)	CESTtoNPI	0.042	0.001	0.083	0.0462	0.124
12	log(deaths) ~ CESTtoNPI + log(popEstimateNearestThousand)	log(popEstimateNearestThousand)	0.167	-0.257	0.591	0.4340	0.124
13	logEstimatedMaxInt ~ CESTtoNPI	CESTtoNPI	0.043	0.008	0.077	0.0172	0.186
14	logEstimatedMaxInt ~ log(popEstimateNearestThousand)	log(popEstimateNearestThousand)	0.087	-0.355	0.529	0.6890	0.006
15	logEstimatedMaxInt ~ case10toNPI	case10toNPI	0.079	0.023	0.135	0.0075	0.229
16	logEstimatedMaxInt ~ CESTtoNPI * R0 + log(popEstimateNearestThousand)	CESTtoNPI	0.337	0.131	0.543	0.0024	0.437
16	logEstimatedMaxInt ~ CESTtoNPI * R0 + log(popEstimateNearestThousand)	R0	3.619	1.084	6.155	0.0070	0.437

**16**	logEstimatedMaxInt ~ CESTtoNPI * R0 + log(popEstimateNearestThousand)	log(popEstimateNearestThousand)	-0.12	-0.587	0.347	0.6020	0.437
**16**	logEstimatedMaxInt ~ CESTtoNPI * R0 + log(popEstimateNearestThousand)	CESTtoNPI:R0	-0.135	-0.231	-0.038	0.0082	0.437

nCases_maxNPI: number of reported cases at the time of the maximum non-pharmaceutical intervention (NPI).
CESTtoNPI: number of days between the central epidemic seeding time (CEST) and the maximum non-pharmaceutical intervention (NPI).
popSize: census population size for the site under study.
case10toNPI: number of days between the tenth reported case and the maximum non-pharmaceutical intervention (NPI).
Ne: viral effective population size at the time of the maximum non-pharmaceutical intervention (inferred using skygrowth model).
R0: basic reproduction number, inferred either in an SEIJR model (in regression model 16) or using skygrowth (in regressions model 8).
Deaths: number of deaths one month after the maximum non-pharmaceutical intervention.
EstimatedI: number of estimated infections at the time of the maximum non-pharmaceutical intervention, inferred in an SEIJR model.

Supplementary Table 2 | Results from the BEAST analysis for 30 sites.

Site analysed	BEAST R0	Estimated infections at max NPI	Sample Proportion at max NPI (%)
Abruzzo, Italy	1.64	29.6	0
Ahmedabad, India	2.46	143.8	25.03
Antwerp, Belgium	1.84	1155.8	8.11
Auvergne Rhone Alpes, France	2.54	16666	1.44
Basque Country, Spain	1.88	6845	6.65
Comunitat Valenciana, Spain	2.16	6601.9	6.7
Denmark	2.25	1525.6	52.7
Ghent, Belgium	2.45	4295.5	1.78
Hauts de France, France	2.46	4744.5	4.45
Ile de France, France	2.97	18982.8	6.54
Illinois, USA	1.79	13146.5	5.71
Innsbruck, Austria	2.3	2063.9	4.92
Jerusalem District, Israel	1.83	615.3	7.15
King County, WA, USA	2.35	11324.4	10.3
Kinshasa, DRC	2.17	1878.5	10.81
Luxembourg	2.37	5743.8	3.85
Madinah, Saudi Arabia	2.72	3172.9	0.03
Makkah, Saudi Arabia	1.53	328.7	47.16
Munich, Germany	2.09	2240.3	1.76
NewOrleans, LA, USA	4.18	6055.1	7.45
Portugal	2.79	1868.9	42
St. Petersburg, Russia	1.88	2893	1.73
San Diego, CA, USA	2.23	11248.2	0.93
San Francisco, CA, USA	2.13	7724.6	0.48
South Coast District, Israel	2.2	1589.7	2.77

Switzerland	2.28	3950.1	55.69
Taipei, Taiwan	1.76	2795.6	1.57
Telangana, India	3.28	525.8	5.14
Tel Aviv District, Israel	2.09	1450.3	4.21
Wisconsin, USA	2.29	6802.2	6.15

Note: R0 and the number of infections at the time of the maximum non-pharmaceutical intervention (max NPI) were inferred in an SEIJR model using BEAST. Sample proportion was calculated at max NPI as the number of reported infections divided by the number of estimated infections.

Supplementary Figures

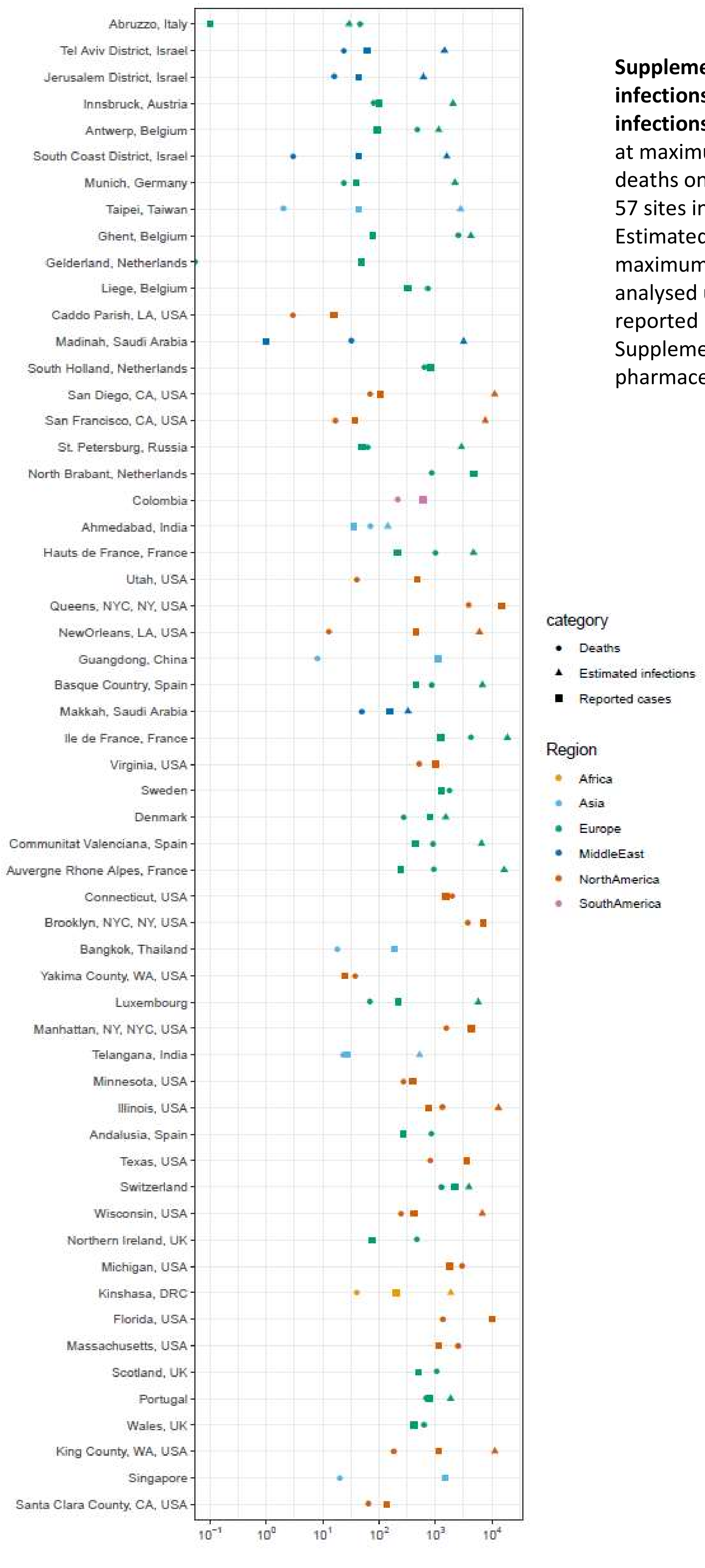

Supplementary Figure 1 | Reported infections and deaths and estimated infections. Reported number of infections at maximum NPI, reported number of deaths one month after maximum NPI for 57 sites included in the analysis. Estimated number of infections at maximum NPI are displayed for 30 sites analysed using BEAST. Sources for reported numbers are available in Supplementary Data 1. NPI: Non-pharmaceutical intervention.

Supplementary Figure 2 | Locations of all 57 sites included in the analyses, denoting the number of SARS-CoV-2 genetic sequences available for each site. These numbers are also available in Supplementary Data 1. CEST: Central Epidemic Seeding Time; NPI: Non-pharmaceutical intervention. This map was created with ggmap[21] using a Natural Earth map, available to use without permission.

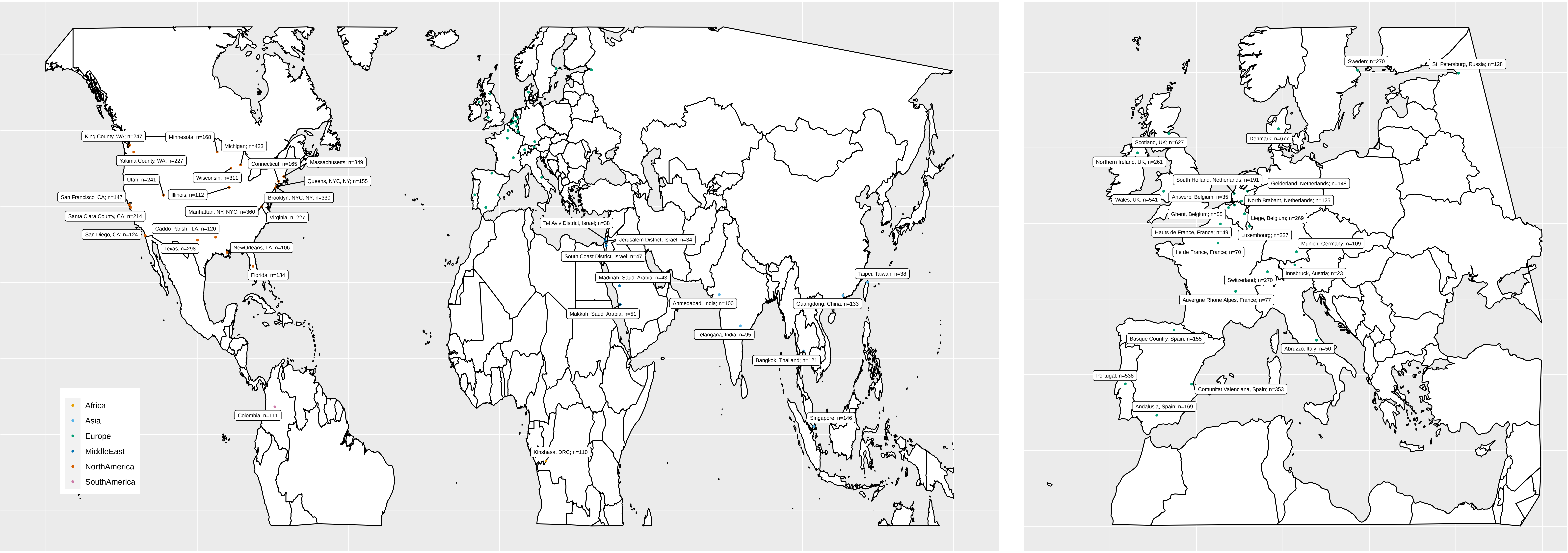
King County, WA; n=247
Minnesota; n=168
Michigan; n=433
Yakima County, WA; n=227
Connecticut; n=165
Massachusetts; n=349
Wisconsin; n=311
Utah; n=241
Queens, NYC, NY; n=155
San Francisco, CA; n=147
Illinois; n=112
Brooklyn, NYC, NY; n=330
Santa Clara County, CA; n=214
Manhattan, NY, NYC; n=360
Virginia; n=227
Caddo Parish, LA; n=120
San Diego, CA; n=124
Texas; n=298
NewOrleans, LA; n=106
Florida; n=134
Colombia; n=111
Tel Aviv District, Israel; n=38
Jerusalem District, Israel; n=34
South Coast District, Israel; n=47
Madinah, Saudi Arabia; n=43
Makkah, Saudi Arabia; n=51
Ahmedabad, India; n=100
Telangana, India; n=95
Taipei, Taiwan; n=38
Guangdong, China; n=133
Bangkok, Thailand; n=121
Singapore; n=146
Kinshasa, DRC; n=110
Africa
Asia
Europe
MiddleEast
NorthAmerica
SouthAmerica
Sweden; n=270
St. Petersburg, Russia; n=128
Denmark; n=677
Scotland, UK; n=627
Northern Ireland, UK; n=261
South Holland, Netherlands; n=191
Gelderland, Netherlands; n=148
Antwerp, Belgium; n=35
North Brabant, Netherlands; n=125
Wales, UK; n=541
Ghent, Belgium; n=55
Liege, Belgium; n=269
Hauts de France, France; n=49
Luxembourg; n=227
Munich, Germany; n=109
Ile de France, France; n=70
Innsbruck, Austria; n=23
Switzerland; n=270
Auvergne Rhone Alpes, France; n=77
Basque Country, Spain; n=155
Abruzzo, Italy; n=50
Portugal; n=538
Comunitat Valenciana, Spain; n=353
Andalusia, Spain; n=169

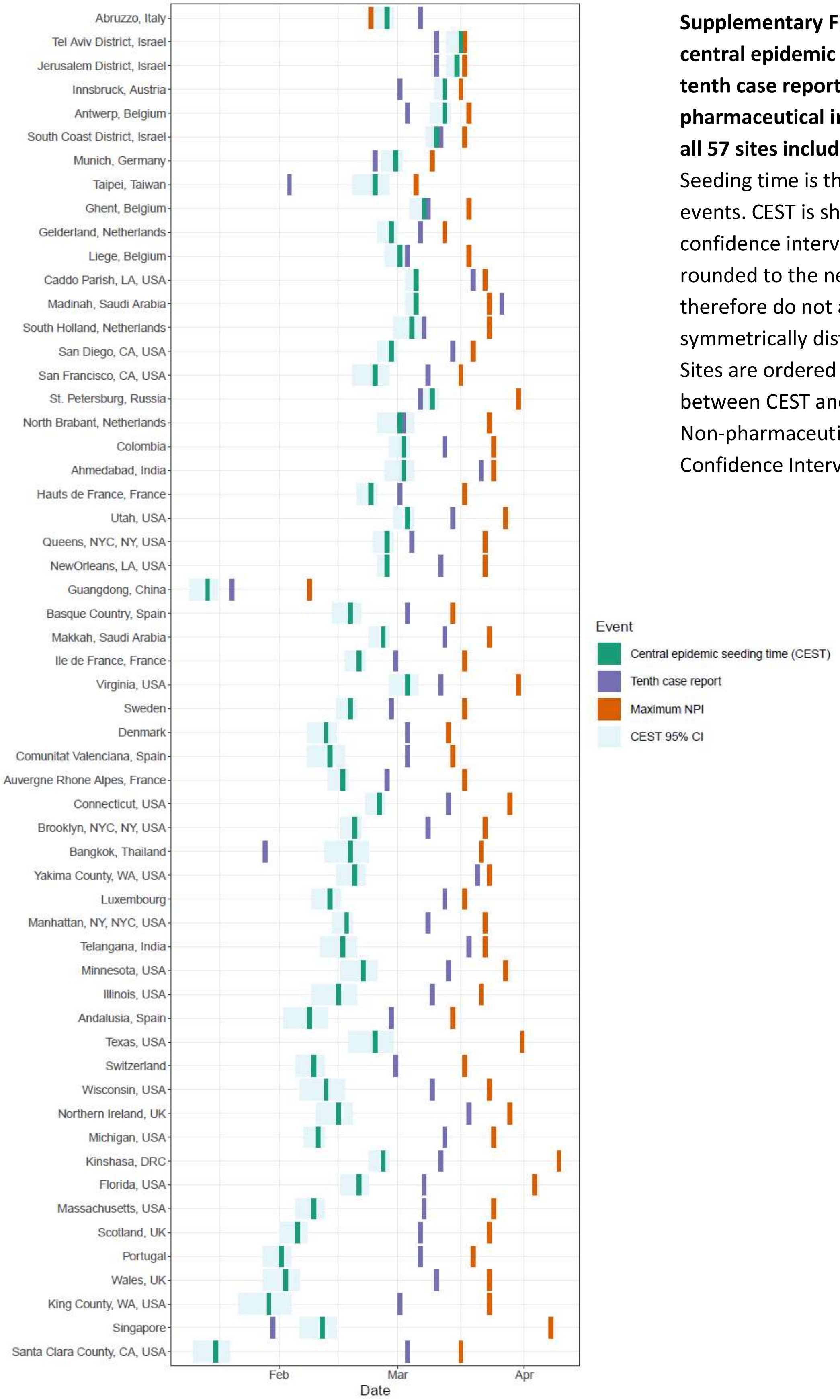

Supplementary Figure 3 | Dates of central epidemic seeding time (CEST), tenth case report and maximum non-pharmaceutical interventions (NPI) for all 57 sites included in our analysis. Seeding time is the time of importation events. CEST is shown with 95% confidence intervals (CI). 95% CI are rounded to the nearest day and therefore do not always look symmetrically distributed around CEST. Sites are ordered by the duration of time between CEST and maximum NPI. NPI: Non-pharmaceutical intervention; CI: Confidence Interval

Supplementary Figure 4 | Relationship between different measures of epidemic scale and the estimated time to first and maximum NPI. A) Reported diagnosed infections versus time to max NPI. B) Phylodynamic estimates of cumulative infections at time of max NPI versus time to the first NPI. C) Deaths one month after max NPI versus time to the first NPI. D) Viral effective population size (Ne) versus time to the first NPI. E) Viral effective population size (Ne) at max NPI versus reported diagnosed cases at time of max NPI. F) Viral effective population size (Ne) at max NPI versus deaths one month after maximum NPI. NPI: Non-pharmaceutical intervention.

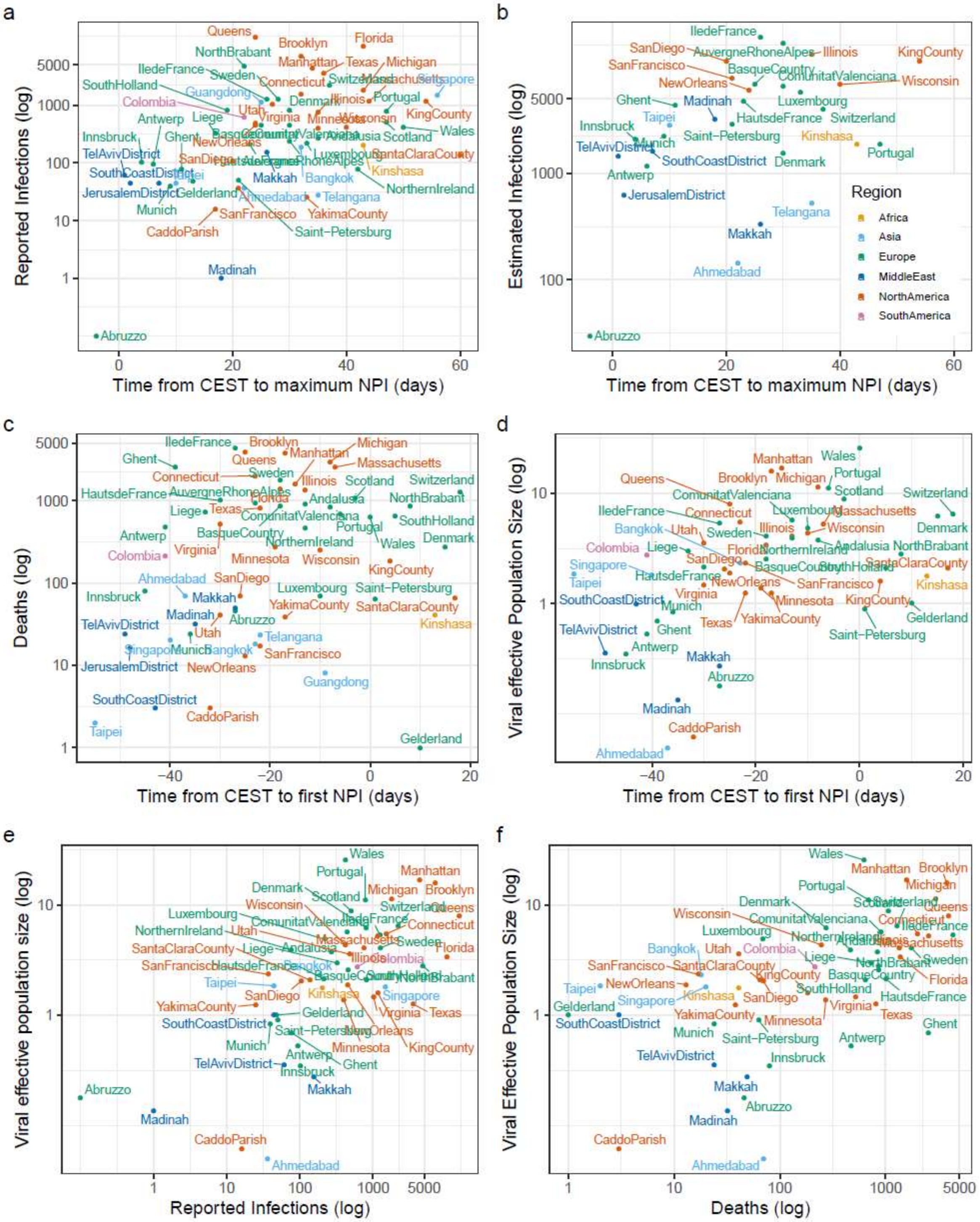

Supplementary Figure 5 | Deming regression of epidemic severity and viral effective population size over estimated time to maximum NPI. A) Cumulative deaths within a month following max NPI. B) Viral effective population size versus time to max NPI. The sizes of circles are inversely proportional to the error in measurement of variables on both the x and y axes. The Deming regression takes into account these errors in measurement when calculating relationships. The slope of the relationship (mean fitted values) and its 95% confidence interval are shown in grey. NPI: Non-pharmaceutical intervention.

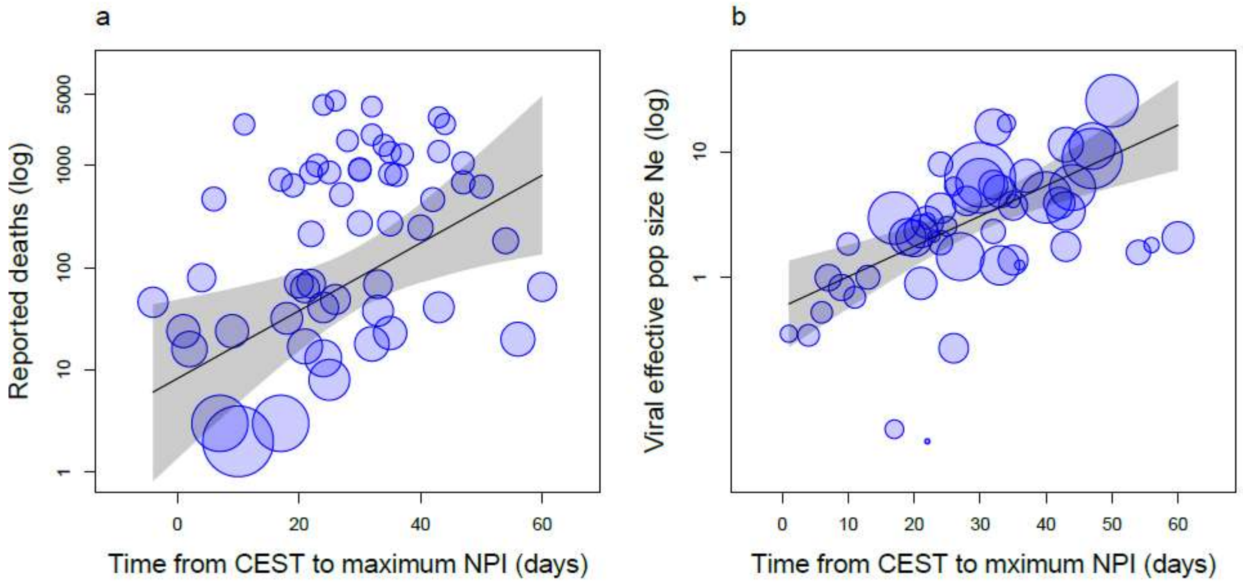

Supplementary Figure 6 | Cumulative estimated infections, estimated daily infections and effective reproduction number through time (R_t) for 30 locations analysed in BEAST. Reported infections for each location are shown using yellow dots, while estimated infections are represented by black lines with 95% highest posterior density estimates in grey. The last sample from each location is indicated by a vertical line of each graph, estimates beyond that time point are projections from the SEIJR model rather than phylodynamic reconstructions.

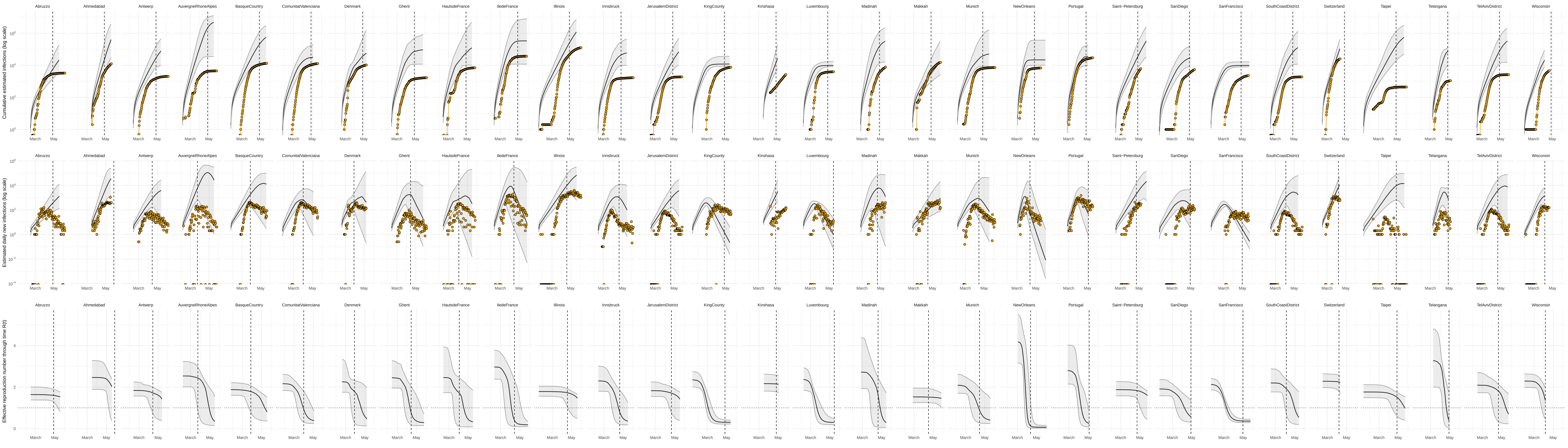
Abruzzo
Ahmedabad
Antwerp
AuvergneRhoneAlpes
BasqueCountry
ComunitatValenciana
Denmark
Ghent
HautsdeFrance
IledeFrance
Illinois
Innsbruck
JerusalemDistrict
KingCounty
Kinshasa
Luxembourg
Madinah
Makkah
Munich
NewOrleans
Portugal
Saint−Petersburg
SanDiego
SanFrancisco
SouthCoastDistrict
Switzerland
Taipei
Telangana
TelAvivDistrict
Wisconsin
Cumulative estimated infections (log scale)
Estimated daily new infections (log scale)
Effective reproduction number through time R(t)
March
May

Supplementary Figure 7 | Correlation between the effective reproduction number R_t (in purple) and changes in mobility over time. Mobility estimates were obtained from Google and measured in relative deviations from maximum mobility prior to the WHO pandemic declaration. Mobility data were not available for Kinshasa, DRC.

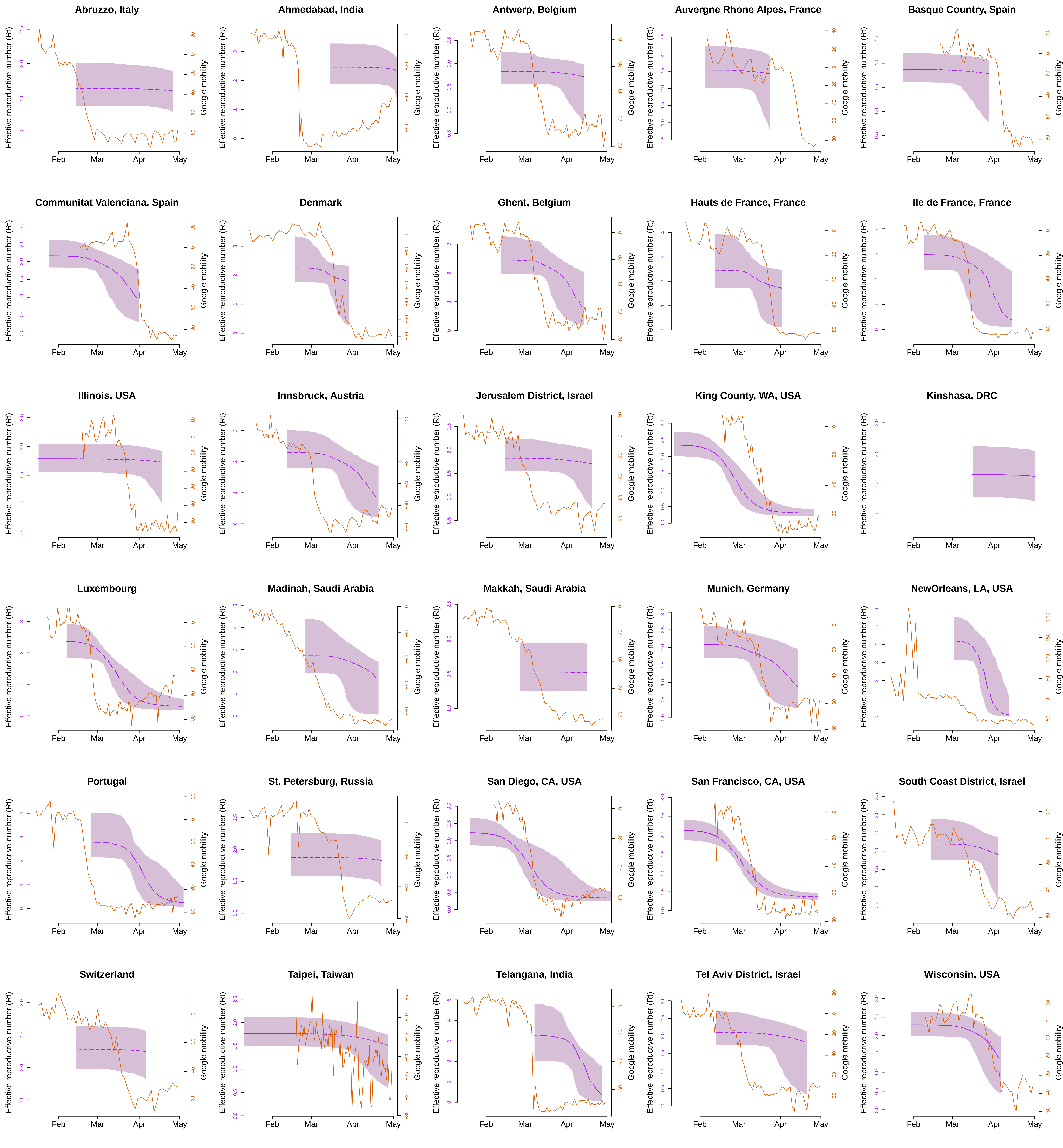
Abruzzo, Italy
Ahmedabad, India
Antwerp, Belgium
Auvergne Rhone Alpes, France
Basque Country, Spain
Communitat Valenciana, Spain
Denmark
Ghent, Belgium
Hauts de France, France
Ile de France, France
Illinois, USA
Innsbruck, Austria
Jerusalem District, Israel
King County, WA, USA
Kinshasa, DRC
Luxembourg
Madinah, Saudi Arabia
Makkah, Saudi Arabia
Munich, Germany
NewOrleans, LA, USA
Portugal
St. Petersburg, Russia
San Diego, CA, USA
San Francisco, CA, USA
South Coast District, Israel
Switzerland
Taipei, Taiwan
Telangana, India
Tel Aviv District, Israel
Wisconsin, USA
Effective reproductive number (Rt)
Google mobility
Feb
Mar
Apr
May

Supplementary Figure 8 | Cumulative estimated infections, estimated daily infections and effective reproduction number through time (R_t) for five simulated datasets. Analysis results are shown each time for the dataset analysed as whole and in deduplicated form. True (simulated) values are shown using yellow dots and reconstructed estimates are shown as black lines with 95% highest posterior density estimates.

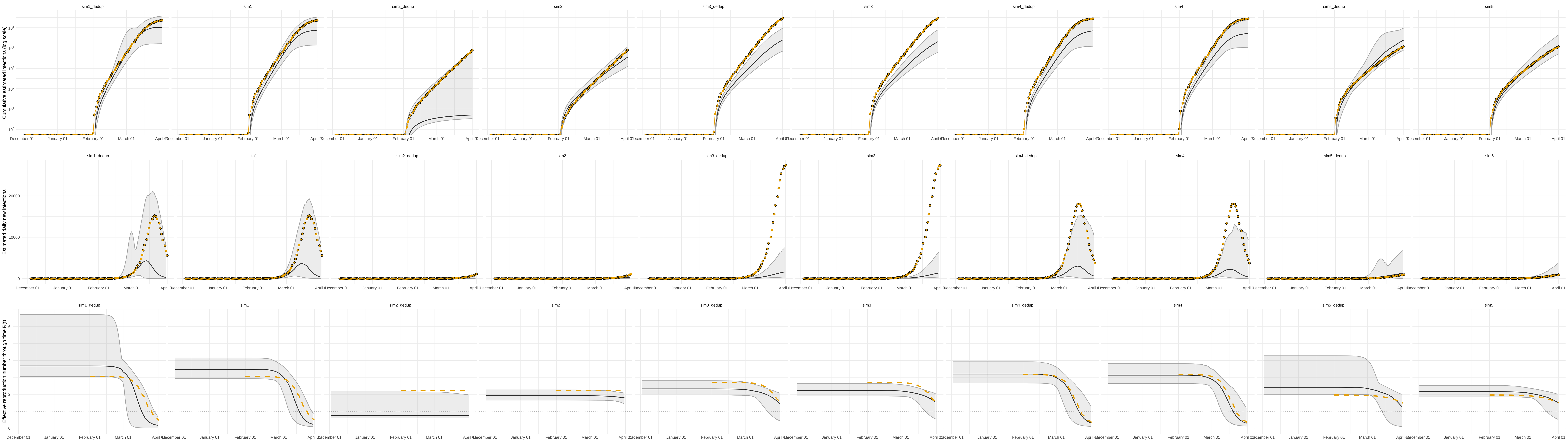
sim1_dedup
sim1
sim2_dedup
sim2
sim3_dedup
sim3
sim4_dedup
sim4
sim5_dedup
sim5
Cumulative estimated infections (log scale)
10^0
10^1
10^2
10^3
10^4
10^5
Estimated daily new infections
0
10000
20000
Effective reproduction number through time R(t)
0
2
4
6
December 01
January 01
February 01
March 01
April 01

Supplementary Methods

1. Overview of data processing pipeline

A. Genetic data cleaning and preparation

SARS-CoV-2 sequences were downloaded from GISAID (gisaid.org) on June 7th 2020. Sequences were removed if they were not sampled from human hosts or if sampling dates were not exact (day/month/year). The Coronavirus Disease Genomics UK Consortium (COG-UK) was launched in March 2020 with the aim of sequencing 10% of all COVID diagnoses[1]. As a result, the proportion of sequences originating from the UK increased dramatically from this date. Disproportional representation of sequences from different places is known to bias phylodynamic and phylogeographic models[2,3], thus we dropped 80% of sequences collected from the UK after March 15th. UK sequences were dropped at random. Remaining sequences were aligned using MAFFT v7[4]. After alignment, we removed sequences with >20% of nucleotide sites missing and cut sequences to the beginning of the first and the end of the last open reading frames. To eliminate badly aligned sequences and sequences problematic for time-resolved phylogenetic analyses, we performed an additional round of data cleaning. We split our data randomly into small subsets (~2,000 sequences) to accelerate the cleaning process. To each subset we added a set of sequences uniformly-distributed through time to improve the resolution of time within the subset. Up to 4 sequences were selected at random for each day in the time period from the first SARS-CoV-2 sample (24/12/2019) to the last sample date, totalling 565 unique sequences spanning the time period. We constructed maximum likelihood phylogenies for each subset using IQtree v1.6[5], and dropped sequences considered to be outliers. Outliers were defined as sequences a) with a mean cophenetic distance >= 3 standard deviations from the mean phylogeny cophenetic distance or b) that did not conform to the molecular clock based on a time-scaled analysis with *treedater* v0.5.0[6]. We included only unique sequences in our final analysis; where replicate sets existed, we removed all but the earliest sequence. All data cleaning was performed in R v3.6.1.

B. Inclusion/ exclusion of sites

Sites were eligible for analysis if there were at least 100 hundred sequences available from that location on GISAID on June 7th (n=77). Fourteen sites with fewer sequences were also analysed, for reasons explained below. Among the 91 sites, we then excluded sites for the following reasons. Our model requires samples to be collected at random across a population and with a range of dates that enables reconstruction of a molecular clock. We excluded locations where samples were known to have been collected as a result of contact tracing or where travellers had been preferentially sequenced (n=8) [7]. Unfortunately, that

information was unavailable for many sites. We chose to exclude identical sequences in case they resulted from contact tracing; but this choice introduces a different kind of bias, as groups of identical sequences are a feature of early rapidly spreading epidemics[8]. Fortunately, in our simulations, exclusion of identical sequences from different individuals did not overly bias results (see below). When data were available for sites located within each other (e.g. New Orleans in Louisiana), the smaller geographic unit was preferentially selected (n=20 removed), and some regions were excluded because they were too large geographically to fit our model assumption of random mixing, given known different dynamics across the region (n=3). One exception to the former rule is Valencia, which was analysed as "Comunitat Valenciana" because labeling of the latter was more systematic (n=1 removed). Wuhan and Hubei were not analysed because we could not have estimated viral origin without including non-human samples (n=2 removed). Fourteen sites with <100 sequences were analysed because these regions were among the first on GISAID to have at least 20 sequences available. Fifty-seven sites were included in our final analysis. Details of inclusion/ exclusion and sample sizes for each site are displayed in Supplementary Table 1.

C. Calculation of variance for Deming regression models

The Deming regression models require estimation of the error for predictor and outcome variables, captured by their variance. Our predictor variables related to time, and included the time from CEST to the first NPI, time from CEST to maximum NPI and time from the tenth case reported to maximum NPI. All variables were expressed in number of days.

Variance of CEST was computed from a bootstrap distribution of 100 phylogenies. Estimated standard deviation (expressed in the same units as the variable) ranged from 0.3 to 1.7 days. In the case of the time delay between tenth case and maximum NPI, a fixed standard deviation of 1 and 2 days were evaluated. For calculating variance on the number of deaths, we modelled observations as a binomial process with sampling frequency p=1% (based on the approximate infection fatality rate for SARS-CoV-2 [9]). For case counts, we modeled observation as a Poisson process (variance of the error of the observation equal to the observation) as a conservative starting point. As the relationship remained significant even with this extreme variance, we did not evaluate further definitions. Viral effective population size through time was extracted from the nonparametric skygrowth model. The number of infections were estimated in the Bayesian SEIJR model in BEAST. In both cases, parameters are estimated from a distribution of trees therefore variance in effective population size and in estimated infections could be calculated directly from those distributions.

2. Phylodynamic model for infectious disease dynamics: Susceptible-Exposed-Infected (IJ)-Recovered (SEIJR)

Terminology: The phylodynamic model is designed to estimate epidemiological parameters using a combination of sequence data from a *region* (e.g. a city, county or other small territory) and *exogenous* sequences from a much larger international reservoir.

Essential metadata: Location (region or exogenous) and date of sampling.

Mathematical model: The mathematical model is based on previous development of SEIR-type models for Ebola virus[10] and implemented in a structured coalescent framework in the PhyDyn package[11]. A related model was applied in the early stage of the SARS-CoV-2 epidemic to estimate global case numbers[12] and has also been applied in studies of local Chinese[13] and Israeli[14] SARS-CoV-2 sequence data. The phylodynamic model is designed to account for

- Nonlinear epidemic dynamics in the region,
- A realistic distribution of generation times with incubation and infectious periods,
- Migration of lineages between region and exogenous demes; and
- Variance in transmission rates which has a large influence on epidemic size estimates.

The model of epidemic dynamics within a region is based on an SEIR model. We elaborate this model with an additional compartment *J* which has a higher transmission rate (τ-fold higher) than the I compartment. Upon leaving the incubation period individuals progress to the J compartment with probability p_h, or otherwise to I.

The model is implemented as a system of ordinary differential equations:

$$\dot{S}(t) = -\left(\beta I(t) + \beta\tau J(t)\right)\frac{S(t)}{S(t)+E(t)+I(t)+J(t)+R(t)}$$
$$\dot{E}(t) = \left(\beta I(t) + \beta\tau J(t)\right)\frac{S(t)}{S(t)+E(t)+I(t)+J(t)+R(t)} - \gamma_0 E(t)$$
$$\dot{I}(t) = \gamma_0(1-p_h)E(t) - \gamma_1 I(t)$$
$$\dot{J}(t) = \gamma_0 p_h E(t) - \gamma_1 J(t)$$
$$\dot{R}(t) = \gamma_1(I(t) + J(t))$$

Parameters: β is the per-capita transmission rate. τ is the ratio of transmission rate in the high to low risk categories. γ_0 is the rate of progression from incubating individuals to infectious individuals (note that this does not describe which individuals are symptomatic). γ_1 is the rate of recovery once infectious.

We also model an exponentially growing (rate ρ) reservoir $Y(t)$ for imported lineages into the *region*.

Migration is modeled as a bidirectional process which only depends on the size of variables in the region compartment and thus migration does not influence epidemic dynamics; it will only influence the inferred probability that a lineage resides within the region.

For a compartment X (E,I, or J), η is the per lineage rate of migration out of the region and the total rate of migration in and out of the region is ηX.

Supplementary Table 3: Parameters and priors of the SEIJR model.

Parameter	Symbol	Prior
Initial infected	E_0	Exponential(1)
Initial susceptible	S_0	Exponential[1]
Migration rate	η	Exponential(10)[2]
Reproduction number	R_0	Lognorm(0.88, sd log=0.5)
Clock rate	ω	Uniform(.0005,.005)[3]
Transition/transversion	κ	Lognorm(1, sd log=1.25)

1. Prior mean for susceptible population was calibrated to individual locations based on population size.
2. Units: Migrations per lineage per year. Maximum value = 10.
3. Units: Substitutions / site / year

During phylodynamic model fitting β and ρ are estimated. Additionally, we estimate initial sizes of Y, E, and S. Other parameters are fixed based on prior information. We fix $1/\gamma_0 = 5$ days and $1/\gamma_1 = 3$ days. Parameters controlling overdispersion in transmission rates (p_h and τ) are estimated with strong priors which yields a dispersion of the reproduction number that matches a negative binomial distribution with $k = 0.22$ if $R_0 = 2$, similar to values estimated for the 2003 SARS epidemic[15].

3. SEIJR phylodynamic model reconstruction of simulated epidemics

A. Simulation of epidemics under the SEIJR model

In order to evaluate the ability of our SEIJR model to reconstruct phylodynamic history and estimate epidemic parameters, we simulated epidemics with known parameters. Thirty combinations of parameters were sampled from the uniform distributions shown in Supplementary Table 4 using latin hypercube sampling as implemented in the lhs R package[16]. Other parameters were fixed (Supplementary Table 4).

From the 30 combinations of parameter values, we calculated the cumulative number of infections for each using phydynR v.0.2.0[17]. We then selected five sets of parameters that displayed a diversity of outcomes (different numbers of cumulative infections, as well as showing recent decreases). These parameters are shown in Supplementary Table 5.

We next simulated phylogenetic trees based on the structured coalescent using the function *sim.co.tree* in phydynR. Tips in the phylogenies trees belonged to two different compartments: regional (I, n=100) and exogenous (*Y*, n=50). The seeding time of the epidemic as a whole was set to the beginning of December (2019.92), the seeding time for the regional epidemic was sampled from a uniform distribution (Supplementary Table 4). Sampling began in January 2020 (2020.0).

Sequences were simulated from the phylogenies using seq-gen[18], as implemented in the phyclust R package[19]. We used an Hasegawa-Kishino-Yano (HKY) DNA substitution model[20] with a transition/transversion rate of 5.5, a clock rate of 0.001 nucleotide substitutions/ year, and relative base frequencies for the frequency of nucleotides A, C, G and T of 0.3, 0.2, 0.2 and 0.3, respectively. Sequences generated were 29,500 bases. We then used a customized function in R to deduplicate the DNA sequence alignment. All scripts to reproduce our simulations are available on github (github.com/thednainus/sarscov2simulations).

B. Analysis of simulated data using BEAST2

Five test datasets for analysis were generated using simulation (for parameters of each, see Supplementary Table 5), and each was analysed in totality and in deduplicated form. Each dataset was processed in BEAST2 using the SEIJR model and parameter priors as described in Supplementary Table 3 to generate the effective

reproduction number R_0, the reproduction number through time, R_t, and an estimated number of infections . True values for daily and cumulative infections fell within 95% highest posterior density (HPD) estimates for 3 out of 5 simulations (Supplementary Figure 6, simulations are labelled 1 to 5). For simulations 3 and 4 estimates of the number infected were below the true value. R_0 true values fell within 95% estimated HPD for R_0 in 4 of 5 simulations. Longitudinal estimates of R_t were within the 95% HPD in 3 out of simulations. In 4 out of 5 simulations R_t was set to decrease and that drop was captured in all 4 of the reconstructions within +/- 1 week of the true drop. De-duplication of the data based on sequence identity did not bias results but tended to increase HPD. Timings of decrease in R_t were unaffected by deduplication. The accuracy of our reconstructions was not diminished with higher rates of import (see simulation 2).

Supplementary Table 4. List of parameters that were sampled from a uniform distribution or fixed in our simulations. *U* denotes Uniform distribution. All rate parameters have units of 1/year.

**Parameters**	**Values**
Transmission rate β	U(15, 25)
Initial number of susceptible individuals	U(1,0000, 1,000,000)
Importation rate η	U(1, 10)
Start time for sampling in the region	U(2020.10, 2020.15)
Initial number of exposed individuals	U(1, 30)
Transmission risk ratio τ	Fixed at 74
Proportion high-risk p_h	Fixed at 0.2
Exogenous growth rate	Fixed at 25
Rate of disease progression γ_0	Fixed at 73
Rate of recovery γ_1	Fixed at 121.667

Supplementary Table 5. List of randomly generated parameters values used to simulate phylogenetic trees.

Simulation number	β	R_0	Initial susceptible	η	Regional epidemic start	Initial E
1	23.93111	3.07	272824.10	5.236837	2020.137	19.661442
2	17.38683	2.23	840389.11	9.113708	2020.111	2.882301
3	21.13614	2.71	596454.58	1.931835	2020.103	25.039326
4	24.69453	3.16	309257.92	1.727232	2020.106	29.417566
5	15.28835	1.96	47565.01	7.214645	2020.140	22.193182

Supplementary Notes

A. Impact of time from CEST to maximum NPI on number of reported infections

Among the 57 sites, CEST to maximum NPI was significantly associated with the number of reported infections at maximum NPI in the Deming (p<0.0001) and the univariate linear regression model (p<0.0001, Sup Tab 1, Sup Fig 2). This time delay explains over a quarter of the variance in the reported number of infections at time of maximum NPI (R^2=0.27) in the linear model, An additional 14 days of transmission before maximum NPI was associated with a 2.13 (95%CI: 1.51-3.00) fold increase in number of reported infections in the Deming model and a 2.88 (95%CI: 1.80-4.66) fold increase in the linear model. The time delay from the tenth reported case to maximum NPI was also predictive of the number of reported infections but explained less of its variance (R^2=0.24, p=0.0001). Census population size was a significant predictor of reported infections in univariate models (R^2=0.17, p=0.0013), but when included alongside time from CEST in multivariable models, it was no longer significant.

B. Impact of time from CEST to maximum NPI on number of estimated infections

Among the 30 sites analysed using the Bayesian phylodynamic model, we generated estimates of the basic reproduction number R_0, R_t and the number of infections over time (Sup Fig 1, 3 and 4, Sup Tab 1 and 2). The time

from CEST to maximum NPI was associated with the model-based estimates of the number of infections at maximum NPI (R^2=0.19, p=0.0172; Sup Fig 2) in the linear regression model but not in the Deming model (p=0.05). Based on the univariate regression, an additional 14 days of transmission before maximum NPI was associated with a 1.82 (95%CI: 1.11-2.94) fold increase in the number of estimated infections at time of maximum NPI. In the multivariable linear model (R^2 =0.44), time to maximum NPI remained significant (p=0.0024). The estimated basic reproduction number R_0 was also significant (p=0.0070) as R_0 is co-inferred alongside the number of infections in the model. A 25% increase in R_0 was associated with a 1.31 (95%CI: 1.80-4.67) fold increase in the number of estimated infections at maximum NPI. The estimated number of infections was correlated with the number of deaths at maximum NPI (Pearson's r=0.39, p=0.032) and with the number of reported infections at that point (Pearson's r=0.64, p=0.0002). The estimated number of infections was highly correlated with viral effective population size (Pearson's r=0.67, p=0.0001), but estimates of R_0 were not (p=0.06). Using phylodynamic estimates of the cumulative number infected we estimated that the mean reporting rate (proportion of infections diagnosed) at the time of maximum NPI was 11.1% but varied greatly between regions and over time (Sup Tab 3, Sup Fig 7).

C. Impact of time from CEST to first NPI on number of reported deaths

Among the 57 sites, time from CEST to first NPI was predictive of the number of deaths one month later in the Deming regression (p=0.0006, Sup Fig 2) and in the univariate linear model (R^2=0.12, p=0.0008). An additional 14 days of transmission before maximum NPI was associated with a 2.58 (95%CI: 1.46-4.56) fold increase in the number of reported infections in the Deming model and a 1.79 (95%CI: 1.17-2.73) fold increase in the linear model.

D. Impact of time from CEST to first NPI on viral effective population size

Time from CEST to first NPI was highly predictive of viral effective population size at the time of maximum NPI in both the Deming model (p=0.0003; Sup Fig 2) and the univariate linear regression (R2=20.80, p<0.0001).

E. Impact of seeding time definitions

As a sensitivity analysis on our definition of the seeding time for each regional epidemic, weo calculated the 5th and 25th percentiles of the distribution of viral introduction times and recalculated the delay to maximum NPI for each definition, as well using CEST. We then calculated the number of days between those two seeding times and the maximum NPI and used the Deming and linear regression model to relate the time from seed to maximum NPI on a) the number of reported deaths one month after NPI, b) viral effective

population size at maximum NPI, c) the number of reported infections at maximum NPI and d) the number of estimated infections at maximum NPI.

Results remained highly consistent with those from our analysis using CEST (Supplementary Table 6). We focused on CEST within the main results because 1) it normalises for different patterns of viral introduction over time across locations: some places had some very early introductions that did not seed many infections, but others did not 2) it ensures that descendant viruses have contributed to the local epidemic.

Supplementary Table 6. Deming and linear regression models with different definitions for seeding time of regional epidemics

**Model**		**Deming regression**		**Linear regression**		
**Predictor**	**Outcome**	**slope**	**p value**	**estimate**	**p value**	**R2**
**q25 to max NPI**	**Deaths**	0.048	0.0081	0.030	0.01	0.073
**q25 to max NPI**	**Ne**	0.034	0.0023	0.036	0.00005	0.273
**q25 to max NPI**	**Reported infections**	0.050	0.023	0.022	0.0006	0.193
**q25 to max NPI**	**Estimated infections**	0.035	<0.0001	0.032	0.01	0.201
**q5 to max NPI**	**Deaths**	0.037	0.0045	0.027	0.03	0.079
**q5 to max NPI**	**Ne**	0.029	0.0008	0.026	0.0008	0.198
**q5 to max NPI**	**Reported infections**	0.029	<0.0001	0.041	0.0006	0.192
**q5 to max NPI**	**Estimated infections**	0.048	0.0249	0.029	0.01	0.192

q5 to max NPI: Time from 5th percentile of viral introduction distribution to maximum NPI
q25 to max NPI: Time from 2th percentile of viral introduction distribution to maximum NPI
Deaths: deaths one month after maximum NPI
Ne: viral effective population size at max NPI
Reported infections: at max NPI
Estimated infections: in BEAST model, at max NPI.

Supplementary References

1. COVID-19 Genomics UK (COG-UK) consortiumcontact@cogconsortium.uk. An integrated national scale SARS-CoV-2 genomic surveillance network. *Lancet Microbe* **1**, e99–e100 (2020).

2. Hall, M. D., Woolhouse, M. E. J. & Rambaut, A. The effects of sampling strategy on the quality of reconstruction of viral population dynamics using Bayesian skyline family coalescent methods: A simulation study. *Virus Evol* **2**, vew003 (2016).

3. De Maio, N., Wu, C.-H., O'Reilly, K. M. & Wilson, D. New Routes to Phylogeography: A Bayesian Structured Coalescent Approximation. *PLoS Genet.* **11**, e1005421 (2015).

4. Katoh, K., Misawa, K., Kuma, K.-I. & Miyata, T. MAFFT: a novel method for rapid multiple sequence alignment based on fast Fourier transform. *Nucleic Acids Res.* **30**, 3059–3066 (2002).

5. Nguyen, L.-T., Schmidt, H. A., von Haeseler, A. & Minh, B. Q. IQ-TREE: a fast and effective stochastic algorithm for estimating maximum-likelihood phylogenies. *Mol. Biol. Evol.* **32**, 268–274 (2015).

6. Volz, E. M. & Frost, S. D. W. Scalable relaxed clock phylogenetic dating. *Virus Evol* **3**, (2017).

7. Seemann, T. *et al.* Tracking the COVID-19 pandemic in Australia using genomics. *Nature Communications* **11**, 4376 (2020).

8. Lu, J. *et al.* Genomic Epidemiology of SARS-CoV-2 in Guangdong Province, China. *Cell* **181**, 997–1003.e9 (2020).

9. Russell, T. W. *et al.* Estimating the infection and case fatality ratio for coronavirus disease (COVID-19) using age-adjusted data from the outbreak on the Diamond Princess cruise ship, February 2020. *Euro Surveill.* **25**, (2020).

10. Volz, E. & Pond, S. Phylodynamic analysis of Ebola virus in the 2014 Sierra Leone epidemic. *PLoS Curr.* **6**, (2014).

11. Volz, E. M. & Siveroni, I. Bayesian phylodynamic inference with complex models. *PLoS Comput. Biol.* **14**, e1006546 (2018).

12. Volz, E., Baguelin, M., Bhatia, S., Boonyasiri, A. & Cori, A. Report 5: phylogenetic analysis of SARS-CoV-2. *allcatsrgrey.org.uk* (2020).

13. Geidelberg, L. *et al.* Genomic epidemiology of a densely sampled COVID19 outbreak in China. *Virus Evolution* (2021).

14. Miller, D. *et al.* Full genome viral sequences inform patterns of SARS-CoV-2 spread into and within Israel. *Nature Communications* **11**, 5518 (2020).

15. Lloyd-Smith, J. O., Schreiber, S. J., Kopp, P. E. & Getz, W. M. Superspreading and the effect of individual variation on disease emergence. *Nature* **438**, 355–359 (2005).

16. Carnell, R. lhs: Latin hypercube samples. *R package version 0. 10, URL http://CRAN. R-project. org/package= lhs* (2012).

17. Volz, E. *phydynR*. (Imperial College London).

18. Rambaut, A. & Grassly, N. C. Seq-Gen: an application for the Monte Carlo simulation of DNA sequence evolution along phylogenetic trees. *Comput. Appl. Biosci.* **13**, 235–238 (1997).

19. Chen, W.-C. Phylogenetic Clustering with R package phyclust. *URL http://thirteen-01. stat. iastate. edu/snoweye/phyclust* **54** (2010).

20. Hasegawa, M., Kishino, H. & Yano, T. Dating of the human-ape splitting by a molecular clock of mitochondrial DNA. *J. Mol. Evol.* **22**, 160–174 (1985).

21. Kahle, D., Wickham, W. ggmap: Spatial Visualisation with ggplot2. *The R Journal* **5**, 144-161 (2013).
