# Supplementary material for: Genetic evidence for the association between COVID-19 epidemic severity and timing of non-pharmaceutical interventions
Source: Nat Commun. 2021 Apr 12;12:2188. doi: 10.1038/s41467-021-22366-y (PMC8041850; doi:10.1038/s41467-021-22366-y)
Supplement: Supplementary file 3 — Descriptions of Additional Supplementary Files [file 41467_2021_22366_MOESM3_ESM.pdf]

Descriptions of Additional Supplementary Files

Supplementary Data 1
Description: Data and sources of data are shown for all 57 sites included in the skygrowth analysis. The BEAST site and Skygrowth site columns indicate whether or not that site was included in each set of analyses. CEST- Central Epidemic Seeding Time (see Methods). Ne - Viral effective population size

Supplementary Data 2
Description: GISAID IDs used in final analyses.
