# Supplementary material for: Genetic evidence for the association between COVID-19 epidemic severity and timing of non-pharmaceutical interventions
Source: Nat Commun. 2021 Apr 12;12:2188. doi: 10.1038/s41467-021-22366-y (PMC8041850; doi:10.1038/s41467-021-22366-y)
Supplement: Supplementary file 6 — Reporting Summary [file 41467_2021_22366_MOESM6_ESM.pdf]

nature research

Corresponding author(s): Manon Ragonnet-Conin

Last updated by author(s): Feb 25, 2021

Reporting Summary

Nature Research wishes to improve the reproducibility of the work that we publish. This form provides structure for consistency and transparency in reporting. For further information on Nature Research policies, see our Editorial Policies and the Editorial Policy Checklist.

Statistics

For all statistical analyses, confirm that the following items are present in the figure legend, table legend, main text, or Methods section.

n/a	Confirmed	
☐	☒	The exact sample size (n) for each experimental group/condition, given as a discrete number and unit of measurement
☐	☒	A statement on whether measurements were taken from distinct samples or whether the same sample was measured repeatedly
☒	☐	The statistical test(s) used AND whether they are one- or two-sided<br>*Only common tests should be described solely by name; describe more complex techniques in the Methods section.*
☐	☒	A description of all covariates tested
☐	☒	A description of any assumptions or corrections, such as tests of normality and adjustment for multiple comparisons
☒	☐	A full description of the statistical parameters including central tendency (e.g. means) or other basic estimates (e.g. regression coefficient) AND variation (e.g. standard deviation) or associated estimates of uncertainty (e.g. confidence intervals)
☐	☒	For null hypothesis testing, the test statistic (e.g. F, t, r) with confidence intervals, effect sizes, degrees of freedom and P value noted<br>*Give P values as exact values whenever suitable.*
☐	☒	For Bayesian analysis, information on the choice of priors and Markov chain Monte Carlo settings
☒	☐	For hierarchical and complex designs, identification of the appropriate level for tests and full reporting of outcomes
☐	☒	Estimates of effect sizes (e.g. Cohen's d, Pearson's r), indicating how they were calculated

Our web collection on statistics for biologists contains articles on many of the points above.

Software and code

Policy information about availability of computer code

Data collection	No software was used for data collection
Data analysis	R v3.6.1; IQtree v1.6; sarscov2 R package v0.1.4 (https://github.com/emvolz-phylodynamics/sarscov2Rutils); BEAST2 v6.1; MAFFT v7; treedater v 0.5.0; skygrowth v0.3.1; tn93 (https://github.com/tn93); Tracer v1.7.1; https://github.com/thednainus/sarscov2simulations (DOI: 10.5281/zenodo.4559446); PhyDyn v1.3.7 (https://github.com/mrc-ide/PhyDyn)

For manuscripts utilizing custom algorithms or software that are central to the research but not yet described in published literature, software must be made available to editors and reviewers. We strongly encourage code deposition in a community repository (e.g. GitHub). See the Nature Research guidelines for submitting code & software for further information.

Data

Policy information about availability of data

All manuscripts must include a data availability statement. This statement should provide the following information, where applicable:

- Accession codes, unique identifiers, or web links for publicly available datasets
- A list of figures that have associated raw data
- A description of any restrictions on data availability

All sequence data are freely available upon registration with the GISAID database (https://www.gisaid.org) . Dates of Data sources for case and death numbers are available in the Supplementary Data. Dates of lockdown were extracted from the Oxford COVID-19 Government Response Tracker (downloaded 20/06/2020; Hale, T., Webster, S., Petherick, A., Phillips, T. & Kira, B. Oxford covid-19 government response tracker. Blavatnik School of Government 25, (2020).)

Field-specific reporting

Please select the one below that is the best fit for your research. If you are not sure, read the appropriate sections before making your selection.

☐ Life sciences ☐ Behavioural & social sciences ☒ Ecological, evolutionary & environmental sciences

For a reference copy of the document with all sections, see nature.com/documents/nr-reporting-summary-flat.pdf

Ecological, evolutionary & environmental sciences study design

All studies must disclose on these points even when the disclosure is negative.

Study description	We looked at the relationship between time from SARS-CoV-2 viral introduction and lockdown, and different measures out epidemic severity, including case numbers, deaths, number of estimated infections and viral effective population size.
Research sample	Sites were eligible for analysis if there were at least 100 hundred sequences available from that location on GISAID on June 7th (n=78). Fourteen sites with fewer sequences were also analysed, for reasons explained below. Among the 82 sites, we then excluded sites for the following reasons. Our model requires samples to be collected at random across a population and with a range of dates that enables reconstruction of a molecular clock. We excluded locations where samples were known to have been collected as a result of contact tracing or where travellers had been preferentially sequenced (n=8) (5,6) . Unfortunately, that information was unavailable for many sites. We chose to exclude identical sequences in case they resulted from contact tracing; but this choice introduces a different kind of bias, as groups of identical sequences are a feature of early rapidly spreading epidemics(7) . Fortunately, in our simulations, exclusion of identical sequences from different individuals did not overly bias results (see below). When data were available for sites located within each other (e.g. New Orleans in Louisiana), the smaller geographic unit was preferentially selected (n=21), and some regions were excluded because they were too large geographically to fit our model assumption of random mixing (n=3). One exception to the former rule is Valencia, which was analysed as "Communitat Valenciana" because labeling of the latter was more systematic. Wuhan and Hubei were not analysed because we could not have estimated viral origin without including non-human samples. Fourteen sites with <100 sequences were analyzed because these regions were among the first on GISAID to have at least 20 sequences available. Fifty-seven sites were included in our final analysis. Details of inclusion/ exclusion and sample sizes for each site are displayed in the Supplementary Data.
Sampling strategy	Our sample was a convenience sample based on data made publicly available and which fit our requirements and assumptions,a s listed above. Through trial and error, we established that our SEIJR model performed well if at least 100 sequences were available. Because our sample was a convenience sample, dependent on data shared with GISAID, we started testing our models early in the epidemic on locations as soon as 20 sequences were available. Fourteen sites were thus included with <100 sequences for historical reasons, because we processed them early in the epidemic, with successful convergence of BEAST runs.
Data collection	GISAID data were downloaded and processed by MRC twice a week, to generate a list of sites with sufficient sequence data for phylodynamic analysis. This processing was conducted in R v3.6.1. Additional data for each site selected (n=57) were manually extracted from public databases, as listed in the Supplementary Data.
Timing and spatial scale	On June 7th 2020, with over 50 independent locations with >100 sequences available, we determined that the GISAID database comprised a sufficient amount of data and number of sites for our analysis to be feasible. Until that date, we has been downloading GISAID data once a week and counting the number of sequences available for each independent location. On June 7th, the most recent sample in the GISAID database dated from May 30th, thus sample dates included in this study ranged from 2020-01-08 to 2020-05-30. Samples originated from 57 locations (24 in Europe, 20 in North America, five in the Middle East, six in Asia, one in South America and one in Africa). The location of our sites was not decided by the authors, but rather based on publicly available data in GISAID, labeled by location.
Data exclusions	We excluded sites for the following reasons. Our model requires samples to be collected at random across a population and with a range of dates that enables reconstruction of a molecular clock. We excluded locations where samples were known to have been collected as a result of contact tracing or where travellers had been preferentially sequenced (n=8) (5,6) . Unfortunately, that information was unavailable for many sites. We chose to exclude identical sequences in case they resulted from contact tracing; but this choice introduces a different kind of bias, as groups of identical sequences are a feature of early rapidly spreading epidemics (7) . Fortunately, in our simulations, exclusion of identical sequences from different individuals did not overly bias results (see below). When data were available for sites located within each other (e.g. New Orleans in Louisiana), the smaller geographic unit was preferentially selected (n=21), and some regions were excluded because they were too large geographically to fit our model assumption of random mixing (n=3). One exception to the former rule is Valencia, which was analysed as "Communitat Valenciana" because labeling of the latter was more systematic. Wuhan and Hubei were not analysed because we could not have estimated viral origin without including non-human samples.
Reproducibility	Our regression analyses were repeated with multiple outcomes: deaths, case numbers, estimated infections and viral effective population size. Multiple regression models were used: ordinary linear regression and Deming regression
Randomization	This study was an observational study, conducted on a convenience sample based on publicly deposited data, and thus randomisation was not possible.
Blinding	Our analysis did not include allocating data into groups, therefore blinding was not applicable.

Did the study involve field work? ☐ Yes ☒ No

Reporting for specific materials, systems and methods

We require information from authors about some types of materials, experimental systems and methods used in many studies. Here, indicate whether each material, system or method listed is relevant to your study. If you are not sure if a list item applies to your research, read the appropriate section before selecting a response.

Materials & experimental systems

n/a	Involved in the study
☒	☐ Antibodies
☒	☐ Eukaryotic cell lines
☒	☐ Palaeontology and archaeology
☒	☐ Animals and other organisms
☒	☐ Human research participants
☒	☐ Clinical data
☒	☐ Dual use research of concern

Methods

n/a	Involved in the study
☒	☐ ChIP-seq
☒	☐ Flow cytometry
☒	☐ MRI-based neuroimaging
